# Intravitreal faricimab for treatment naïve patients with neovascular age-related macular degeneration: a real-world prospective study

**DOI:** 10.1186/s40942-024-00586-w

**Published:** 2024-09-30

**Authors:** Gabriela Grimaldi, Giuseppe Cancian, Arianna Paris, Michele Clerici, Giulio Volpe, Moreno Menghini

**Affiliations:** Department of Ophthalmology, Institute of Clinical Neurosciences of Southern Switzerland (INSI), Ente Ospedaliero Cantonale (EOC), Via Pietro Capelli 1, Lugano, 6962 Switzerland

**Keywords:** Faricimab, Age-related macular degeneration, Anti-vascular endothelial growth factor, Anti-angiopoietin 2, Treat-and-extend

## Abstract

**Background:**

Intravitreal faricimab, a bispecific antibody targeting both angiopoietin-2 (Ang-2) and vascular endothelial growth factor-A (VEGF-A), was recently introduced for the treatment of neovascular age-related macular degeneration (nAMD), diabetic macular oedema and cystoid macular oedema secondary to retinal vein occlusion. The aim of our study was to assess the efficacy, safety and durability of intravitreal faricimab in a real-world cohort of treatment-naïve patients with nAMD.

**Methods:**

Single-centre, prospective cohort study of 21 eyes from 19 treatment-naïve nAMD patients who were treated with intravitreal faricimab from October 2022 to April 2024. Patients underwent a loading dose (LD) of 4 monthly faricimab injections followed by a treat-and-extend regimen. Primary outcomes included best-corrected visual acuity (BCVA) and structural parameters from spectral-domain optical coherence tomography (SD-OCT). Secondary outcomes included the proportion of eyes achieving a dry macula, maximal fluid-free interval and intended interval at last follow-up.

**Results:**

The study included 21 eyes of 19 patients (mean age 83.1 years). After LD, 93.3% of eyes achieved a dry macular SD-OCT scan within a median time of 8 weeks. At the first extension, 53% of eyes remained dry, while 47% showed fluid recurrence. Long-term analysis (*n* = 14) revealed significant reductions in macular volume (MV), central subfield thickness (CST), and pigment epithelial detachment (PED) height over a median follow-up of 64.9 weeks, with sustained visual and anatomical improvements. Median BCVA, CST, and MV at the final follow-up were significantly improved from baseline (*p* < 0.01). The intended interval between injections was ≥ 12 weeks in 42.86% of eyes. No cases of intraocular inflammation were observed, although 10% experienced retinal pigment epithelial tears.

**Conclusions:**

Intravitreal faricimab demonstrated favourable efficacy, safety, and durability outcomes in a real-world cohort of treatment-naïve nAMD patients.

## Background

Intravitreal anti-vascular endothelial growth factor (anti-VEGF) agents are the standard of care for the treatment of neovascular age-related macular degeneration (nAMD) [1]. In nAMD, the abnormal growth of blood vessels in the macula occurs due to elevated levels of vascular endothelial growth factor-A (VEGF-A), which results in severe visual impairment. Traditional anti-VEGF agents include ranibizumab, bevacizumab and aflibercept, which have proven effective throughout the years in enhancing both visual and anatomical outcomes in patients with nAMD [1]. However, these medications often require frequent intravitreal injections, posing a significant burden on patients and healthcare services. As a result, the focus has shifted towards developing more durable molecules allowing longer intervals between treatments.

Faricimab (Vabysmo, Roche/ Genentech, Basel, Switzerland) was recently introduced in Europe for the treatment of nAMD and diabetic macular oedema, receiving its first authorization for use in Switzerland in May 2022 [2]. Administered intravitreally, faricimab acts through dual-inhibition, targeting both angiopoietin-2 (Ang-2) and VEGF-A [3]. Ang-2 is a growth factor belonging to the angiopoietin/Tie (tyrosine kinase with Ig and EGF homology domains) signalling pathway, which is one of the main pathways involved in angiogenesis. Eyes with pathological conditions such as nAMD have elevated levels of Ang-2, which can exacerbate vascular instability through vascular leakage, inflammation, and neovascularization [4].

TENAYA and LUCERNE were the first phase 3 clinical trials showing the visual benefits obtained with intravitreal faricimab in treatment-naïve patients with nAMD. In both these trials, best-corrected visual acuity (BCVA) change from baseline with faricimab was non-inferior to intravitreal aflibercept, with a comparable rate of adverse events [3]. A key finding from both trials was the durability of treatment effect, as treatment outcomes were achieved with fewer injections in faricimab-treated patients when compared to bimonthly aflibercept. Specifically, 74.1% and 81.2% of faricimab-treated patients received every 12 weeks (Q12W) or longer dosing at week 48 in TENAYA and LUCERNE, respectively, and durability was shown to be further extended in year 2 [5]. Over the past 2 years, several studies have been published evaluating treatment outcomes in real-world cohorts of nAMD patients treated with intravitreal faricimab [6]. Among these, a minority of studies have focused on treatment-naïve patients and favourable outcomes have been reported [7–14].

The aim of our study was to assess the efficacy, safety and durability of intravitreal faricimab in a real-world cohort of treatment-naïve patients with nAMD.

## Methods

This was an observational, single-centre, prospective cohort study of all consecutive treatment-naïve patients with nAMD who were treated with intravitreal faricimab at the Ophthalmology department of Ente Ospedaliero Cantonale in Lugano, Switzerland, from October 2022 to April 2024. Patients were excluded in case of poor quality of retinal imaging or in case of vitreoretinal surgical treatment performed after nAMD diagnosis. The study adhered to the tenets of the Declaration of Helsinki and approval was obtained by the local ethics committee of Canton Ticino (2023 − 00653 CE 4340).

At baseline, demographic data (age, sex) were recorded, and multimodal retinal imaging was performed for each study eye, including ultra-widefield (UWF) colour fundus retinography (Optos PLC, Dunfermline, UK), fluorescein angiography, indocyanine green angiography and spectral-domain optical coherence tomography (SD-OCT) (Heidelberg Engineering, Heidelberg, Germany), to phenotype the macular neovascularization (MNV).

At each appointment, patients underwent the following examinations: best corrected visual acuity (BCVA), intraocular pressure (IOP), and slit-lamp evaluation with dilated fundoscopy. UWF colour retinography and SD-OCT were repeated at each time point during follow-ups.

### Treatment protocol

All patients were treated on label according to the TENAYA and LUCERNE protocols [15], with a loading dose of 4 monthly injections of 6 mg Faricimab followed by a +/- 2-week T&E regimen. Eyes with incomplete anatomical response at week 12, described as the presence of any fluid on structural SD-OCT, were maintained on monthly injections until complete fluid resolution and/or maximal visual improvement were achieved. Conversely, in case of dry macular SD-OCT at week 12, the injection interval was extended by 2 weeks. At subsequent follow-ups, the interval was further extended by 2 weeks in the absence of fluid, whereas in the presence of fluid, the injection interval was modified based on central subfield thickness (CST) change. Specifically, the interval was maintained in case of CST increase equal or less than 10% or reduced by 2 weeks if the CST increase was higher than 10%.

### Treatment outcomes

Primary outcome measures included BCVA and structural SD-OCT parameters. Each SD-OCT image was a macular volume scan of 49 horizontal scans, centred on the fovea and images were acquired in follow-up mode over the study period. Three main SD-OCT parameters were selected for quantification of anatomical efficacy: CST, defined as the mean retinal thickness between internal limiting membrane and Bruch’s membrane (BM) of the circular area within 1 mm diameter around the centre of the fovea; total macular volume (MV), defined as the mean volume of the retina in a circular area within 6 mm diameter around the fovea, and pigment epithelial detachment (PED) height, defined as the longest distance between BM and retinal pigment epithelium (RPE), using the integrated automated segmentation of the Heidelberg Eye Explorer software V.2 (Heidelberg Engineering, V.2.4.1, Heidelberg, Germany) with manual correction of B-scans in case of segmentation errors. These morpho-functional parameters were measured at 4 timepoints: T0, baseline; T1, at the end of loading dose (week 12); T2, after the first extension; T3, at last available follow-up. Secondary outcomes were the ratio of eyes achieving a dry macular SD-OCT scan during the loading phase, the time to a completely dry macular SD-OCT scan, the ratio of eyes displaying a dry macular SD-OCT scan after the first extension to 8 weeks, the maximal fluid-free interval and the intended interval as per the last clinic appointment. In addition, we analysed the proportion of patients that would have been managed differently if disease activity criteria from the pivotal trials TENAYA and LUCERNE [15] were applied at the time of first extension after the loading dose (T2).

### Statistical analysis

Differences in BCVA, CST, macular volume and PED height between timepoints (T0, T1, T2 and T3) were compared using the Wilcoxon Signed-Rank test.

Safety data, clinical characteristics and smoking status were recorded for all patients who started treatment with faricimab and underwent at least 2 faricimab injections. Median time to dry SD-OCT scan and analysis of SD-OCT scan after first extension were calculated for all patients who reached T2. The remaining treatment outcomes were calculated for patients who had a minimum follow-up of 44 weeks. Statistical analysis was performed using RStudio (version 4.4.0). Data distributions were summarised by median, interquartile range (Md [IQR]) and range (minimum–maximum), while proportions were summarized by number of eyes and percentage. BCVA was measured in decimals and for the purpose of this analysis converted to the logarithm of the minimum angle of resolution (logMAR). *P* values inferior to 0.05 were considered statistically significant.

## Results

Twenty-one eyes of 19 patients were included in the study. The most prevalent MNV type at baseline was Type 3 (38.1%) and two patients were smokers (11%). Demographic and baseline clinical data are summarized in Table 1.

**Table 1:** Patient demographics

	Number (*n*)	Ratio (%)
Total number of eyes	21	
Right eyes	12	57
Total patients	19	
Females	16	84
Smokers	2	11
	**Mean ± SD**	**Range**
Age (years)	83.1 ± 6.7	74–99
Clinical characteristics at baseline (*n* = 21)		
	**Number (n)**	**Ratio (%)**
Type 1 MNV	7	33.3
Type 3 MNV	8	38.1
Type 4 MNV	5	23.8
PCV	1	4.8
PED presence	16	76.2
- Flat	3	18.8
- Dome-shaped	7	43.8
- RAP-associated	6	37.5

IVT: intravitreal treatment; IQR: interquartile range; MNV: macular neovascularization; PCV: polypoid choroidal vasculopathy; PED: pigment epithelium detachment; RAP: retinal angiomatous proliferation; SD: standard deviation

Three eyes (14%) had a foveal-involving submacular haemorrhage at baseline, none of which was surgically treated before initiation of treatment with intravitreal faricimab.

### Short-term efficacy

Only eyes with a complete loading dose were included in the analysis of short-term efficacy (15 eyes of 13 patients, 71.4%). The mean age was 84.1 ± 7.4 years and the most prevalent MNV type was Type 3 (6 eyes, 40.0%). Fourteen out of 15 eyes (93.3%) reached a dry macular SD-OCT scan between T0 and T1 and the median time to a dry scan was 8 weeks [IQR 4, 8]. After the loading dose, eyes were extended to a median of 7 weeks [IQR 6, 8]. After first extension (T2), 7 eyes (47%) showed recurrence or persistence of fluid on macular SD-OCT, while 8 eyes (53%) showed a dry macular SD-OCT scan. Four of the eyes with macular fluid at T2 had intraretinal fluid (57%) and 3 had subretinal fluid (43%). Eyes which were extended to 6 weeks or kept at 4 weeks after loading dose (*n* = 8, 53%) displayed a dry macular OCT scan at T2 in 6 cases (75%). Eyes which were extended to 7, 8 or 9 weeks after loading dose (*n* = 7, 47%) displayed a dry macular OCT scan in 2 cases (29%).

At T2, if personalized treatment interval phase dosing criteria from pivotal trials were applied, 10 eyes (67%) would have been treated in the same way while 5 eyes (33%) would have been extended.

### Long-term efficacy and durability

Only eyes with at least 44 weeks of follow-up were included in the long-term efficacy and durability analysis (14 eyes of 12 patients, 67%). Most patients were females (83%) and the mean age was 82.4 ± 7.0 years. At baseline, MNV types were as follows: Type 1, 3 eyes (21%), type 3, 6 eyes (43%); type 4, 4 eyes (29%) and 1 case of PCV (7%).

The median follow-up time was 64.9 weeks [IQR 55.9, 73.1] and the total number of faricimab injections was 10 [IQR 8, 11].

**Fig. 1:**
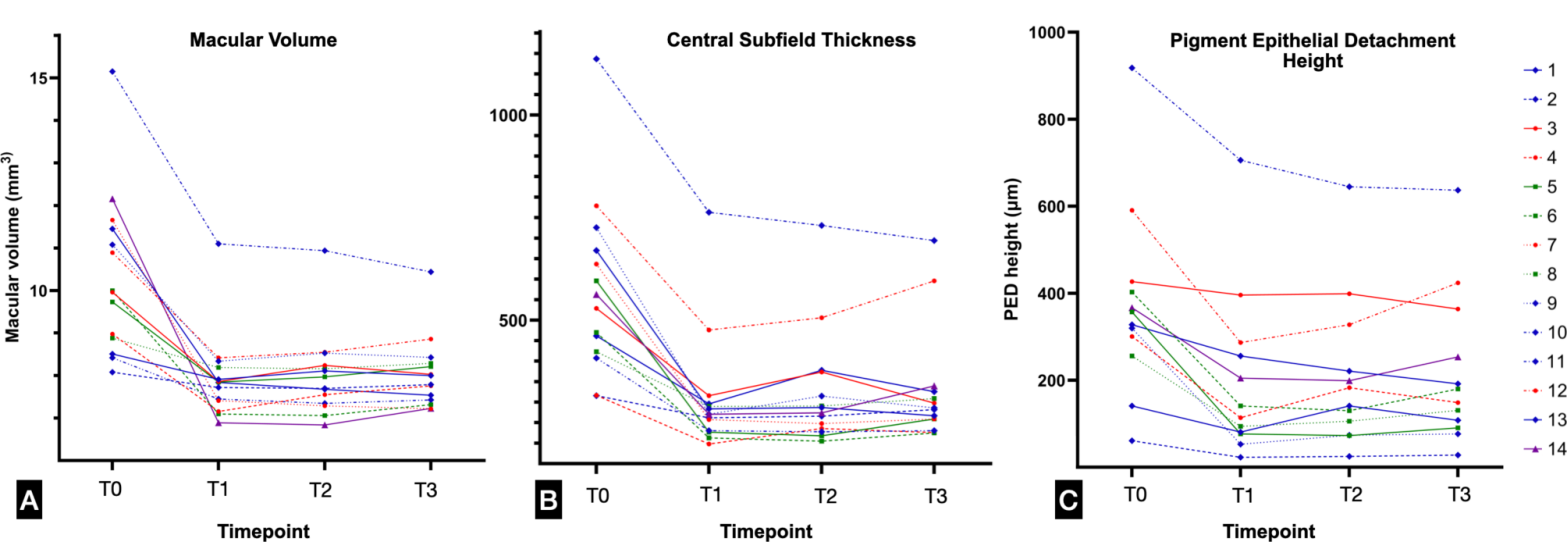
**A-C**. Changes in structural parameters for each study eye (*n* = 14) between timepoints T0 (baseline), T1 (week 12), T2 (post-first extension), T3 (last visit). Neovascularisation subtypes were identified using different colours (Type 3, blue; polypoidal choroidal vasculopathy, purple; Type 1, green; Type 4, red). A rapid and marked improvement in MV, CST and PED height is noted at T1, with mild changes during the maintenance phase (T1-T3). **A**. Macular volume (MV). **B**. Central subfield thickness (CST). **C**. Pigment epithelial detachment (PED) height

Median changes in structural parameters (MV, CST, PED height) between timepoints (T0-T3) are shown in Table 2 and changes for each individual patient are illustrated in Fig. 1.

**Table 2:** Functional and morphological outcomes

	Baseline (T0) Md [IQR]	Week 12 (T1) Md [IQR]	1st Extension (T2) Md [IQR]	Last follow-up (T3) Md [IQR]
BCVA (logMAR)	0.4 [0.3, 0.7]	0.3 [0.1, 0.4]	0.3 [0.1, 0.4]	0.1 [0.1, 0.4]
CST (µm)	546 [423, 664]	271 [231, 296]	281 [236, 374]	284 [259, 326]
Macular volume (mm^3^)	9.98 [8.88, 11.45]	7.85 [7.41, 8.19]	7.84 [7.35, 8.24]	7.90 [7.43, 8.29]
PED height (µm)	343 [279, 415]	128 [79, 272]	162 [90, 275]	165 [100, 309]

BCVA: best corrected visual acuity; CST: Central Subfield Thickness; PED: Pigment Epithelial Detachment

Median MV at week 12 and after the first extension were significantly lower than before treatment (*p* < 0.001). Median MV after first extension was not significantly different from median MV at week 12 (*p* = 0.45). Median MV at last follow-up was significantly lower than MV before treatment (*p* < 0.001), whereas it was not significantly different from MV at week 12 (*p* = 0.18).

Median CST at T1 and at T2 were significantly lower than CST at baseline (*p* < 0.01). Median CST at T2 was not significantly different from median CST at T1 (*p* = 0.25). At last follow-up, median CST was significantly lower than CST at baseline (*p* < 0.001), whereas it did not differ significantly from median CST at T1 (*p* = 0.08).

Median PED height at T1, at T2 and at T3 were significantly lower than PED height before treatment (*p* < 0.01). Median PED height at T2 was not significantly different from median PED height at T1 (*p* = 0.53). Median PED height at last follow-up was not significantly different from median PED at T2 (*p* = 0.31).

Median BCVA at T1 and T2 were significantly improved compared to baseline (*p* < 0.05). Median BCVA at T2 was not significantly different from median BCVA at T1 (*p* > 0.05). Median BCVA at last follow-up was significantly lower than VA at baseline (*p* < 0.01). Median VA at last follow-up was significantly better than median VA at T1 (*p* < 0.05).

Four patients (28.5%) underwent cataract surgery during follow-up (1 case after the 6th, 1 after the 7th and 2 cases after the 9th intravitreal faricimab injection). No change to treatment regimen with intravitreal faricimab was made in these cases, with patients continuing to receive faricimab injections as planned at their last injection clinic appointment.

**Fig. 2:**
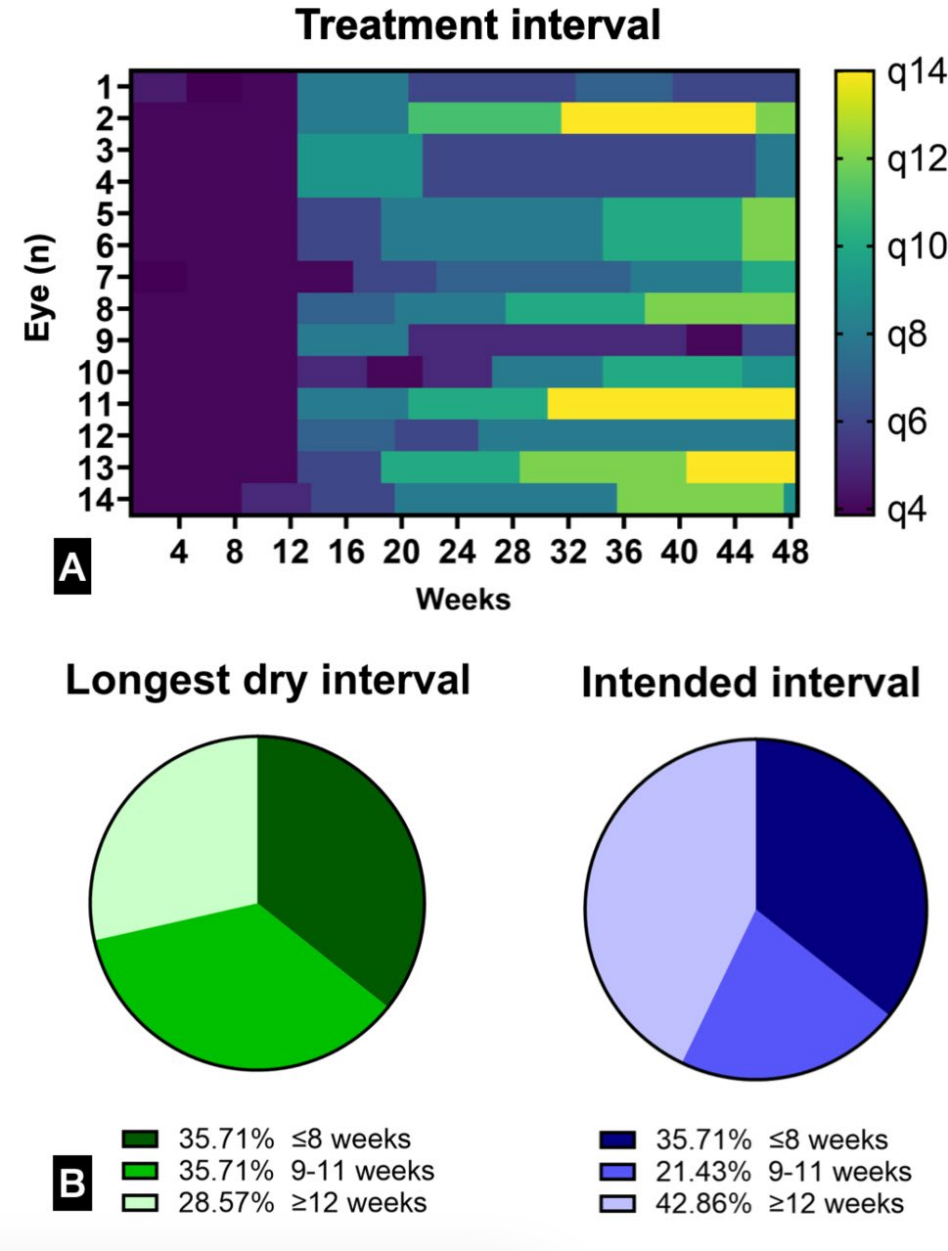
**A-B**. **a**. Heatmap graph showing longitudinal changes in dosing interval trends over 48 weeks. Horizontal lines show trends for each individual case (*n* = 14) over time, with colder and warmer colours representing shorter or longer dosing intervals, respectively. **b**. Pie graphs highlighting the proportion of study eyes achieving a longest dry interval (left, green) or achieving an intended interval at last visit (right, blue) ≤ 8-week dosing, between 9 and 11-week dosing or ≥ 12 weeks as per structural spectral-domain macular optical coherence tomography. The proportion of eyes extended or maintained to ≥ 12 weeks at the last clinic visit was 42.86% although only 28.57% of eyes achieved a longest dry interval ≥ 12 weeks, suggesting fluid tolerance in 14.29% of cases

The distribution of maximal fluid-free dosing intervals and of intended intervals at the last clinic visit among study eyes are shown in Fig. 2, alongside dosing intervals for each individual patient over time. The median time to the second faricimab injection was 4 weeks [IQR 4, 4]. The median time to the third injection was 8.0 weeks [IQR 8.0, 8.3]. The median time to the fourth injection was 12.0 weeks [IQR 12.0, 12.3]. The median time between the fourth and fifth injections was 6.9 weeks [IQR 6.0, 8.0].

After a median follow-up time of 1 year, only 1 patient out of 14 did not display a dry OCT scan (7.1%). The remaining 13 eyes reached a median maximal fluid-free interval of 10 weeks (range 7, 13).

### Safety

Analysis of safety was performed for all eyes who received at least 2 intravitreal injections of faricimab (*n* = 21). No case of intraocular inflammation was observed. Two patients (10%) developed a retinal pigment epithelial (RPE) rip. In both cases, a type 1 MNV was detected at baseline and the RPE rip was visible at week 4 after the first injection. PED height was 469 μm in one case and 555 μm in the second case.

At the last available appointment, 17 out of 21 eyes (81%) were still being treated with intravitreal faricimab. Treatment was suspended in 4 eyes (19%) for the following reasons: 1 case experienced morphological improvement without functional benefit hence treatment was not continued after the third faricimab injection; 1 case was switched to another intravitreal agent after 13 faricimab injections due to recurrence of intraretinal fluid upon extension to every 6 week (Q6W) dosing; 1 patient with unilateral nAMD declined further treatment after completion of the loading dose due to systemic health problems (pancreatic cancer at terminal stage) and 1 patient with unilateral nAMD (4.8%), aged 78, died after week 4 due to exacerbation of heart failure in the context of known ischemic heart disease and atrial fibrillation.

## Discussion

This study evaluated the short-term and long-term efficacy, safety, and durability of intravitreal faricimab for treatment-naïve nAMD patients. To the best of our knowledge, this is the largest real-world, European cohort of naïve nAMD patients treated with faricimab, followed prospectively up to 77 weeks of follow-up.

To date, several real-world studies have been published worldwide detailing experience with intravitreal faricimab in nAMD. Most of these studies assessed changes in morpho-functional outcomes in patients switched to faricimab from traditional anti-VEGF compounds [9, 16–18]. Only a few studies have been published reporting treatment outcomes in the treatment-naïve nAMD population. These studies, which are summarized in Table 3, were conducted predominantly on Asian cohorts over a relatively short follow-up period. Foreseeably, available cohorts in the literature differ from our study in the composition of lesion types, due to the higher prevalence of polypoidal choroidal vasculopathy among Asian populations, which can influence treatment outcomes and generalizability to other populations. With this regard, our study expands the geographic and demographic understanding of faricimab efficacy and safety, showing positive outcomes across cases of type 1, mixed (type 4) and type 3 neovascularisations.

**Fig. 3:**
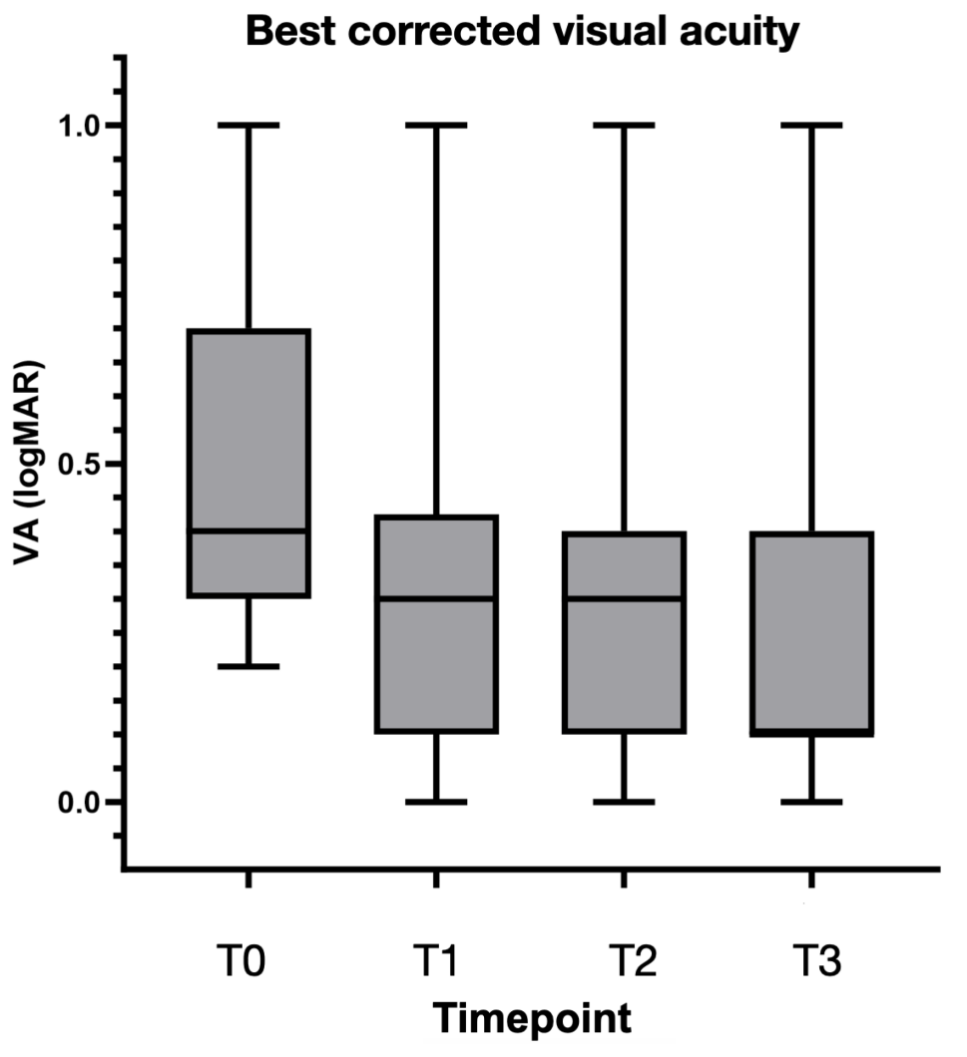
Variations in median best corrected visual acuity (BCVA) between timepoints T0 (baseline), T1 (week 12), T2 (post-first extension), T3 (last follow-up)

It is well known that the success of anti-VEGF therapy in nAMD patients is multifactorial, involving patient-specific characteristics, the nature and management of the disease, and adherence to a structured treatment regimen [19, 20]. While published naïve cohorts received 3 monthly injections of faricimab as loading dose, in our study all patients received at least 4 intravitreal injections of intravitreal faricimab during the loading period, as per label. This difference in the loading dose regimen may have implications for the comparative efficacy and durability of faricimab across studies. Remarkably, our short-term efficacy analysis revealed that 93.3% of the eyes achieved a dry macular SD-OCT scan, within a median time of 8 weeks following the loading dose. This finding is consistent with previous studies, such as those by Matsumoto et al. and Mukai et al., which reported significant improvements in anatomical outcomes after initial treatment with faricimab [7, 10]. However, the recurrence or persistence of fluid in 47% of the eyes, noted in our cohort at T2, highlights the need for individualized treatment regimens. Interestingly, if personalized treatment interval phase dosing criteria from pivotal trials were applied, 67% of our study eyes would have been treated similarly, indicating that although trial protocols seem to be largely applicable to real-world settings, a relevant percentage of patients would still receive a more conservative approach in clinical practice. Nonetheless, it should be noted that our patient population had relatively advanced macular disease on average at baseline, which is reflected in the relatively high baseline MV and CST values. This factor possibly explains to some extent why visual and durability outcomes were less favourable in our series compared to those observed in the pivotal trials, although a positive anatomical trend was observed in all cases irrespective of baseline status (Fig. 1). This result seems to suggest that intravitreal faricimab is overall significantly effective at drying up naïve eyes with nAMD, although a discrepancy with visual trends, where a broader range of outcomes was observed, suggests that rapidity of action and favourable anatomical outcomes alone might not predict a proportional visual recovery in all cases (Fig. 3).

Over follow-up, our study showed sustained efficacy with a significant reduction in structural SD-OCT parameters (MV, CST and PED height) compared to baseline. While improvements in structural parameters mostly occurred between baseline and T1 (i.e. during the loading period), we observed sustained visual acuity gains over longer follow-ups. This result should be interpreted with caution as cataract surgery was performed in 28.5% of cases after T2, acting as a confounding factor for visual trends. In these patients, the delayed improvement in vision may indeed be primarily attributed to the removal of cataracts rather than to the effect of faricimab alone.

When analysing durability, the proportion of eyes whose intended interval at the last available clinic visit was superior or equal to 12 weeks was 42.86% (Fig. 2B). This proportion was larger than the corresponding one on the longest dry interval pie chart (28.57%), suggesting that a few patients were extended to longer dosing intervals despite not being completely dry from a structural standpoint. Overall, durability outcomes were inferior to those reported in pivotal trials TENAYA and LUCERNE, where eyes on extend dosing interval (≥ Q12W) accounted to 79.7% and 77.8% of case, respectively [3]. More favourable outcomes (Q12W = 30%; Q16W = 43.3%) were also observed in the Japanese cohort by Matsumoto et al., which is to date the only other available real-world study assessing nAMD naïve cases with 1-year follow-up [8]. In year 1, patients from the pivotal trials were maintained on fixed intervals ranging from Q8W to Q16W after the loading dose [15]. In our study, the median total number of faricimab injections was relatively high (*n* = 10), although it should be noted that a complete loading dose was performed in all case and that in year 1 the dosing interval was adjusted at each clinic visit following a traditional treat-and-extend regimen, as expected in a real-world clinical setting. Moreover, as previously emphasized, not only did our cases present with more advanced disease at baseline, but we also adopted a more conservative approach to dosing intervals compared to the criteria used in the Tenaya and Lucerne pivotal trials, resulting in shorter intervals between injections. Had we applied the Tenaya and Lucerne criteria, different clinical decisions might have been made, potentially leading to longer dosing intervals and ‘warmer’ heatmap trends (Fig. 2A).

As far as safety is concerned, no case of intraocular inflammation was reported in our series. This represents a more favourable outcome compared to data from pivotal trials and real-world studies, where a small rate of intraocular inflammatory adverse events (0.2–2.5%) was reported [3, 8, 15]. Nonetheless, this discrepancy could be easily explained by differences in patient population and, more importantly, in sample size. Conversely, an opposite trend was the relatively high frequency of RPE tears in our series (*n* = 2, 9%) compared to pivotal trials where 2 cases each (2%) of RPE tears were reported for both TENAYA and LUCERNE cohorts. Other real-world series showed similar results, with 2 cases (3.2%) reported by Mukai et al., 1 case by Tanaka et al. (4.3%) and 1 case by Stanga et al. (33%). The relatively more frequent occurrence of RPE tears in our case series is not unexpected, considering the average clinical severity at baseline in our series, with significantly raised median PED height before treatment.

The primary strengths of this study lie in its prospective design, including deep phenotyping at baseline and thorough structural monitoring for changes over follow-up, as well as a treatment regimen and a patient population that reflect real-world practice. However, the study also has notable limitations. The small sample size limits the generalizability of the results and confounding factors as cataract surgery performed during the maintenance phase in 4 cases cannot be overlooked.

## Conclusions

In conclusion, intravitreal faricimab showed significant efficacy, safety, and durability in a real-world cohort of treatment-naïve European patients with nAMD. By extending treatment intervals and maintaining robust anatomical and visual improvements, faricimab represents a promising advancement in the management of nAMD, addressing some of the key limitations of traditional anti-VEGF therapies. Larger, multicentre studies are warranted to validate these findings and explore the long-term safety and efficacy of faricimab in diverse patient populations. Additionally, investigating the molecular mechanisms underlying the dual inhibition of VEGF-A and angiopoietin-2 could provide deeper insights into optimizing treatment protocols and enhancing therapeutic outcomes.

**Table 3:** Published real-world studies on intravitreal faricimab for treatment naïve neovascular age-related macular degeneration

Study	Eyes	Country	Design	FUP	Total IVT during LD	Outcome measures	PCV rate	IOI rate and management
Matsumoto et al. [7]	40	Japan	Retrospective, monocentric	16 week	3	BCVA, FT, CCT, dry macula rate, regression of polypoidal lesions	18 PCV (17 PCV, 1 Mixed PCV and type 2) (45%)	1 (2,5%) Vitritis without visual loss, treated with subtenon injection of triamcinolone acetonide and betamethasone eye drops
Matsumoto et al. [8]	40 (30 completed 1-year FUP)	Japan	Retrospective, monocentric	1 year	3	BCVA, FT, CCT, total number of IVT, intended injection interval	14 PCV (13 PCV, 1 Mixed PCV and type 2) (46,6%)	1 (2,5%) Vitritis without visual loss, treated with subtenon injection of triamcinolone acetonide and betamethasone eye drops
Khanani et al. [9]	39	USA	Retrospective, multicentric	Post-1 and post-3 IVT	3	BCVA, CST, PED height, rate of eyes with residual exudative changes (IRF, SRF, PED) on OCT, safety		- 1 (0.2%*) infectious endophthalmitis treated with intravitreal antibiotics - 1 (0.2%*) mild anterior chamber inflammation, treated with topical steroids
Mukai et al. [10]	62	Japan	Retrospective, multicentric	3 months	3	BCVA, CFT, SCT, rate of eyes with residual exudative changes (IRF, SRF, PED) and dry macula rate on OCT, regression of polypoidal lesions	22 PCV (35%)	0
Tanaka et al. [11]	23	Japan	Prospective, monocentric	Post-3 IVT	3	BCVA, CRT, CCT, rate of eyes with residual exudative changes (IRF, SRF, PED height, SHRM height) and dry macula rate on OCT, predictive factors (CRT, SHRM height, presence of intact foveal ELM, IRF, SRH)	PCV vs. no PCV, PNV vs. non-PNV 10 PCV (43,5%)	N/A
Stanga et al. [12]	3	UK	Retrospective, monocentric	Post-1 IVT	N/A	BCVA, CRT, FT, rate of eyes with residual exudative changes (IRF, SRF, drusenoid PED) on OCT		N/A
Maruyama-Inoue et al. [13]	47	Japan	Retrospective, monocentric (comparison with 37 eyes injected with brolucizumab)	4 months	3	BCVA, CFT, CCT, rate of eyes with residual exudative changes (IRF, SRF, PED, SHRM) and dry macula rate on OCT	12 PCV (25,5%)	0
Hara et al. [14]	30	Japan	Retrospective, monocentric (comparison with 30 eyes injected with aflibercept)	Post-3 IVT	3	BCVA, CCT, CRT, rate of eyes with residual exudative changes (IRF, SRF, PED) on OCT	15 PCV (50%)	N/A

* Not specified if naïve/pre-treated. Rate of adverse events was therefore calculated over the whole population (*n* = 376). BCVA Best Corrected Visual Acuity; CCT Central Choroidal Thickness; CFT Choroidal Foveal Thickness; CRT Central Retinal Thickness; CST Central Subfield Thickness; ELM External Limiting Membrane; FT Foveal Thickness; FUP Follow-up; IOI Intra Ocular Inflammation; IRF Intraretinal Fluid; IVT Intravitreal injection; LD loading dose; logMAR Logarithm of the minimum angle of resolution; OCT Optical Coherence Tomography; PCV Polypoidal Choroidal Vasculopathy; PED Pigment Epithelial Detachment; PNV Pachychoroid Neovasculopathy; SCT Subfoveal Choroidal Thickness; SHRM Subretinal Hyper-reflective Material; SRF Subretinal Fluid; SRH Subretinal Hemorrhage; UK United Kingdom; USA United States of America

## Data Availability

All data generated or analysed during this study are included in this article. Further enquiries can be directed to the corresponding author.

## Abbreviations

Ang-2: Angiopoietin-2
anti-VEGF: Anti-Vascular Endothelial Growth Factor
BCVA: Best-Corrected Visual Acuity
BM: Bruch’s Membrane
CST: Central Subfield Thickness
IQR: Interquartile Range
logMAR: Logarithm of the Minimum Angle of Resolution
MNV: Macular Neovascularization
MV: Macular Volume
nAMD: Neovascular Age-Related Macular Degeneration
PED: Pigment Epithelial Detachment
PCV: Polypoidal Choroidal Vasculopathy
RPE: Retinal Pigment Epithelium
SD-OCT: Spectral-Domain Optical Coherence Tomography
Q12W: Every 12 Weeks
UWF: Ultra-Widefield
VEGF-A: Vascular Endothelial Growth Factor-A
